# Fournier’s Gangrene in an Elderly Male: A Case Report

**DOI:** 10.7759/cureus.64019

**Published:** 2024-07-07

**Authors:** Shalondria J Sears, Katherine Menendez, Beatriz Cobo Dominguez, Brendan Chernicki, Jessica Caushi, Rebecca Cherner

**Affiliations:** 1 Family Medicine, Florida Atlantic University Charles E. Schmidt College of Medicine, Boca Raton, USA; 2 Family Medicine, Broward Health Medical Center, Fort Lauderdale, USA; 3 Family Medicine, Dr. Kiran C. Patel College of Osteopathic Medicine Nova Southeastern University, Clearwater, USA; 4 Family Medicine, Dr. Kiran C. Patel College of Osteopathic Medicine Nova Southeastern University, Fort Lauderdale, USA

**Keywords:** tissue necrosis, emergent general surgery, genital infection, necrotizing fasciitis (nf), fournier's gangrene (fg)

## Abstract

Fournier's gangrene (FG) is a rapidly progressing necrotizing soft tissue infection of the perineum with potential multiorgan involvement, posing significant mortality risks. This case report highlights the clinical presentation, potential risk factors, and emphasizes the critical necessity of immediate antibiotic therapy and surgical debridement, regardless of the causative agents involved. We also aim to provide new images to better visualize a diagnosis of Fournier’s gangrene. We present the case of a 65-year-old male with a history of self-care neglect, hypertension, and extensive tobacco use. The patient presented to the emergency department exhibiting classical symptoms of systemic illness, necessitating a collaborative diagnostic and therapeutic approach involving various medical specialties including family medicine, urology, general surgery, interventional radiology, infectious disease, pharmacy, intensive care, social service, and palliative care teams. Despite aggressive interventions during his 24-day hospitalization, the patient's clinical condition progressively deteriorated. This case underscores the significance of early detection, timely intervention, and interdisciplinary cooperation in optimizing outcomes for patients with Fournier’s gangrene.

## Introduction

Fournier’s gangrene (FG), a life-threatening necrotizing soft tissue infection, typically of polymicrobial origin, primarily affects the perineum. In males, it commonly involves the scrotum and, less frequently, the testicles. Notably, the blood supply to the testicles originates directly from the aorta, contrasting with the perineal blood supply from the pudendal artery. This infection encompasses the Colles, or superficial perineal fascia, with potential spread to the Buck and Dartos fascia, scrotal, and penile tissue, respectively [1]. The necrosis of tissue is often initiated from dermatological defects in the perineum, anus, or urogenital tract, with idiopathic occurrences being less common [2]. Fulminant Fournier’s can extend from the fascial envelopment of the genitalia throughout the perineum, along Scarpa’s fascia into the abdomen, and occasionally into the thighs via the fascia lata. Necrotizing infection in FG is a complex process involving multiple microorganisms identified within the affected tissues, accompanied by polymorphonuclear cell infiltration and fibrinoid coagulation of nutrient arterioles [3]. This virulent condition typically arises from a combination of pathogens acting synergistically in immunocompromised hosts, with common isolates including gram-negative enteric rods (e.g., *Escherichia coli*), gram-positive cocci (e.g., *Streptococci*), obligate anaerobic bacteria (e.g., *Bacteroides* fragilis and *Clostridium perfringens*), and occasionally fungi such as *Candida* [4].

Individuals diagnosed with FG are predominantly male with a ratio of 10:1, typically aged between 60 and 70 years, and often present with comorbid conditions [5]. Various risk factors contribute to the development of this condition, including diabetes mellitus, alcoholism, malnutrition, smoking, hypertension, malignancy, sodium-glucose cotransporter-2 (SGLT2) inhibitors, immunosuppressive therapy, and organ failure [5, 6]. The characteristic symptoms of FG include severe genital pain, rapidly spreading cellulitis, and systemic toxicity manifestations [2,7]. Prodromal symptoms such as fever and lethargy may precede the onset of perineal edema, intense pain, and tenderness by up to seven days. As the condition progresses, the affected skin over the genitalia may darken, accompanied by purulent discharge and subcutaneous crepitation, while the pain diminishes due to nerve tissue necrosis. However, the skin's appearance may not fully reflect the severity of underlying tissue damage, although a feculent odor can sometimes be detected.

Despite advancements in medical care, mortality rates associated with FG have remained relatively stable over the past 25 years, ranging between 7.5% and 19.8% in recent epidemiological studies [7, 8]. A retrospective analysis of 50 patients revealed a significant difference in hospitalization duration between survivors (median of 26 days) and non-survivors (median of eight days), with a p-value of less than 0.001 [9]. The common causes of death are attributed to severe sepsis, characterized by hypotension, fever, tachycardia, and multi-organ failure [7, 10].

Fournier’s gangrene is diagnosed clinically, emphasizing the importance of prompt identification for improved outcomes, albeit challenging in early stages. Incisional biopsy serves as a confirmatory diagnostic measure. Sepsis workup may reveal pertinent findings such as a left shift on complete blood count (CBC), metabolic acidosis on arterial blood gas (ABG) sampling, and signs of disseminated intravascular coagulation, along with positive urine and blood cultures. Pelvic imaging, particularly computed tomographic (CT) scan, is crucial for assessing the disease extent, given its high sensitivity in delineating involved fascial planes and providing quick and accurate imaging in the context of this life-threatening condition [11, 12].

Early broad-spectrum antibiotic therapy and aggressive surgical debridement significantly reduce mortality [7, 13]. Delay in surgical intervention stands as the most modifiable risk factor linked to mortality and is a crucial step for definitive diagnosis [14, 15]. Particularly in patients exhibiting symptoms of systemic toxicity leading to hemodynamic instability and multi-organ failure, prioritizing aggressive resuscitation over diagnostic procedures is paramount. Pending the results of cultures and sensitivities, initiating empirical medical treatment becomes imperative. This typically involves intravenous (IV) carbapenem (1g Q6-8h meropenem) or piperacillin-tazobactam (3.375g Q6h) in combination with clindamycin (600-900 mg/kg Q8-12h) and vancomycin (15-20 mg/kg Q8-12h) [16]. Concurrently, managing any underlying comorbid conditions is integral to the overall success of appropriate interventions aimed at resolving the disease.

In addition to medical management, radical surgical wound debridement plays a pivotal role, with procedures often repeated until all underlying fascia and subcutaneous tissue necrosis is thoroughly removed [2]. Blunt dissection typically reveals easy separation of tissue planes, aiding in the identification and excision of the diseased tissue. Electrocautery is utilized for extensive disease spread, effectively reducing operative blood loss. Considering the rare nature of FG, there exists a necessity to delve deeper into its presentation, underlying risk factors, and causes to enhance clinical outcomes and guide effective treatment strategies. Hence, our primary objective is to explore the clinical presentation, possible etiology, and associated risk factors in this patient's presentation of FG.

## Case presentation

**Figure 1:**
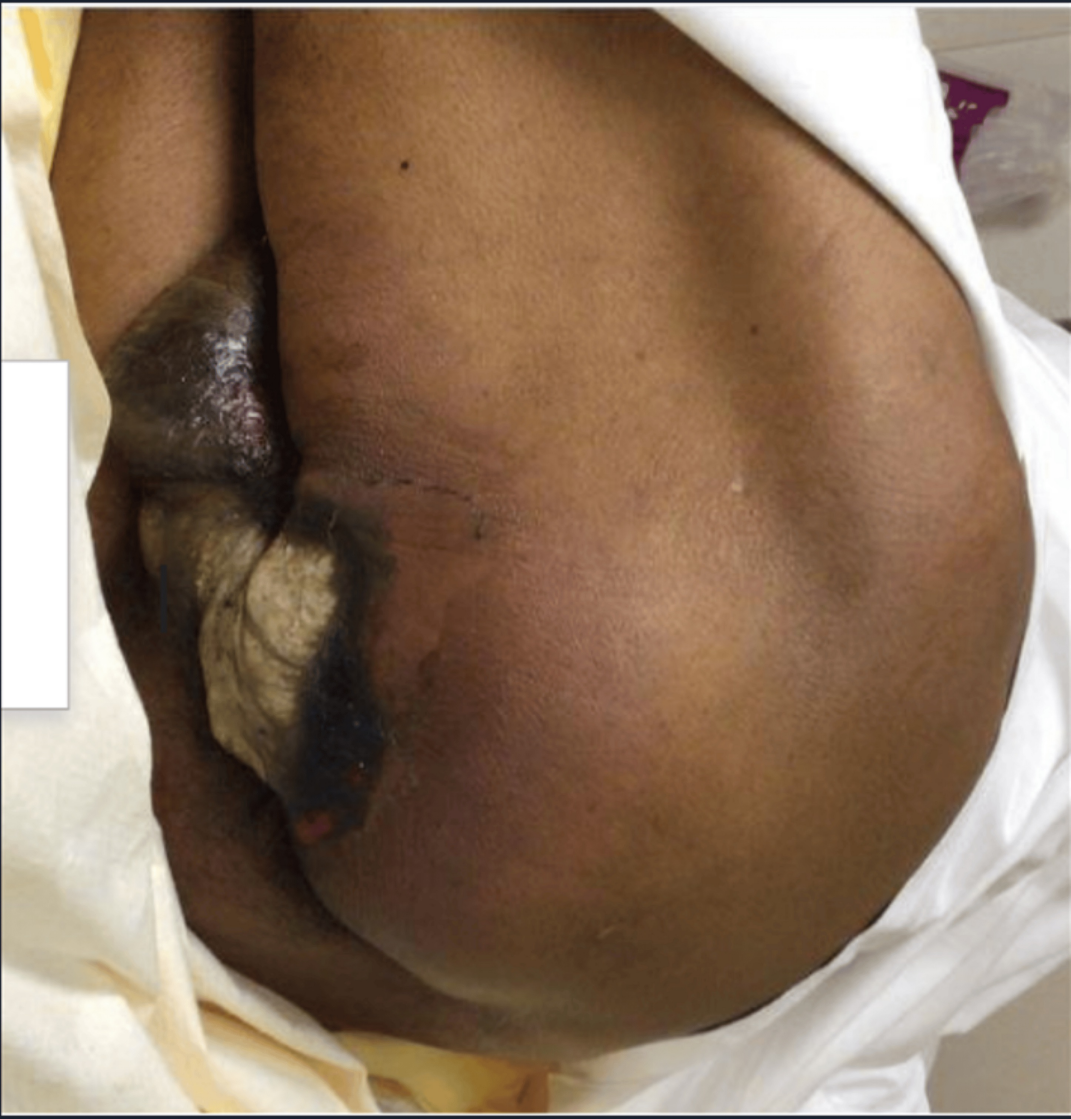
Dry white desquamation of perineum extending into left buttock, erythema and swelling of the testicles.

**Figure 2:**
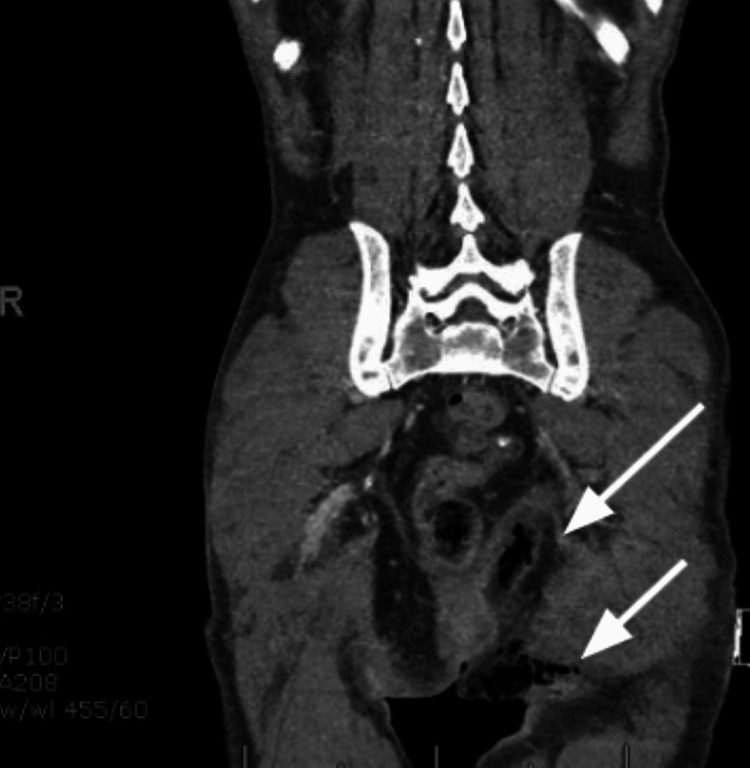
CT abdomen/pelvis shows soft tissue gas locules extending to the left buttock indicated by arrows.

**Figure 3:**
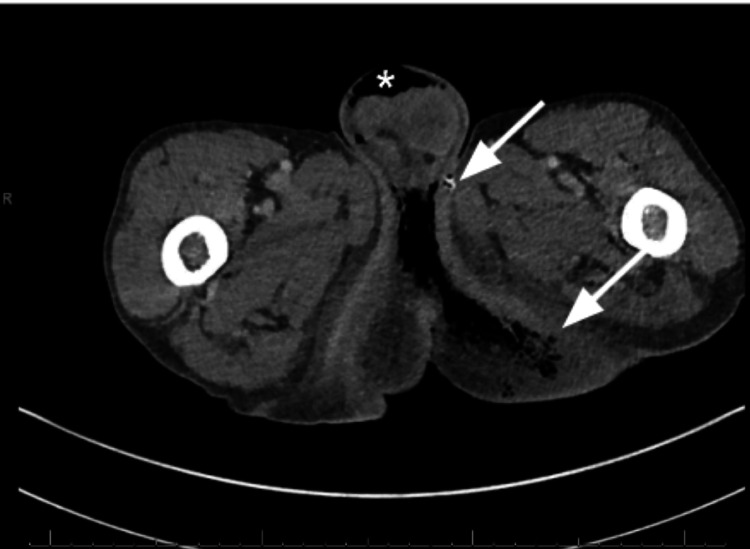
Axial enhanced CT image shows soft tissue edema and fascial thickening (*) in the bilateral scrotum and perineum extending into the left buttock (arrows).

We introduce the case of a 65-year-old male from southeastern Florida, with a past medical history of hypertension, poor self-care, and a 40-pack year history of tobacco use (40 cigarettes/two packs per day over 20 years) who presented to the Emergency Department (ED) with altered mental status of unclear timing. The patient was last seen by a neighbor 10 days prior to admission to the ED. Upon initial evaluation, he was found to be alert and oriented to person but not place or time, following basic commands though was unable to provide significant details about his medical history. Upon examination, the patient was ill-appearing, tachycardic, tachypneic, and hypotensive (98/65 mmHg). Notably, the presence of a foul-smelling odor, hyperpigmentation of the scrotum with dry desquamation, white skin around the buttocks with a small amount of purulent discharge, and palpable crepitus were noted in the ED (Figure 1). Initial labs revealed thrombocytopenia (23,000), hypernatremia (149), high gap metabolic acidosis (14, CO_2_-18), acute kidney injury (AKI) (88:2.1, estimated glomerular filtration rate (eGFR) 41). A peripheral blood smear revealed schistocytes and teardrop-shaped cells. CT abdomen and pelvis with contrast was remarkable for extensive soft tissue air noted along the medial left buttock extending to the perineum and scrotum, compatible with FG (Figures 2 and 3). Furthermore, gas extended into the left ischiorectal fossa and mild gas extended into the right inguinal region. The patient was given lactated Ringer's 1000mL bolus x 2, intravenous (IV) piperacillin-tazobactam 4.5 mg, IV clindamycin 600mg, IV vancomycin 1000 mg, and taken by urology for extensive debridement of the perineum, extending into the anal verge with plans to continue debridement following colostomy creation by general surgery. During the night, the patient experienced multiple episodes of atrial flutter and was subsequently initiated on an amiodarone drip, successfully converting to normal sinus rhythm. However, the patient later exhibited several periods of bradycardia in the early morning. Electrophysiology was consulted for further evaluation and to address concerns regarding a possible atrioventricular (AV) block. Additionally, consultations with infectious disease, nephrology, hematology, and wound care teams were arranged to assist with the comprehensive management of the patient's condition.

Following the initial debridement, the patient required a diverting colostomy before continuing further debridement. Clinically, the patient remained sedated, ventilated, requiring multiple vasopressors, and a foley in the cardiovascular intensive care unit (CVICU). General surgery opted to hold the diverting colostomy due to the patient's progressive pancytopenia unresponsive to replenishment with platelets, fresh frozen plasma (FFP) and IV Immunoglobulin G as well as a developing neutropenia. Tracheal aspirate taken on day 3 exhibited *Candida* infection, whereby micafungin was then added to the pharmacologic regimen. General surgery performed a final extensive debridement of the perineum and perirectal space. Blood and scrotal cultures came back positive for *Escherichia coli*, *Bacteroides fragilis*, and *Proteus mirabilis*, prompting single agent treatment with meropenem, providing adequate coverage for all organisms isolated. The patient remained afebrile over the course of his hospital stay but was ultimately unable to be weaned off of sedation or ventilation. He was given tube feeds with a goal of 50mL/hr with ProSource TF20 60mL daily (1520 kcal, 87 g proteins and 1260mL formula/24 hours). Futhermore, he remained pancytopenic with anoxic bone marrow (WBC 1.85), thrombocytopenic (platelet count 10,000 on day 2 and 6000 on day 3) progressive transaminitis, microangiopathic hemolytic anemia, impending renal failure, and carried an overall poor prognosis. Palliative care was consulted, and the patient was transferred to home hospice care following a family meeting with case management and the patient's next of kin.

## Discussion

Fournier’s gangrene, a rare necrotizing soft tissue infection of the perineum, constitutes a true urological emergency, characterized by a high mortality rate [16, 17]. Typically, FG arises from a localized source of infection, such as a pressure sore or even a seemingly benign infected cut from shaving [18]. This infection swiftly spreads, infiltrating deep fascial planes with distinct obliterative endarteritis, culminating in extensive tissue necrosis and gangrene [19]. The identification of the bacteria through cultures can provide clues to the potential source of infection. Diagnosis relies primarily on clinical assessment, emphasizing the importance of immediate supportive therapy and empiric antibiotic treatment without delay.

Upon presentation, our patient exhibited hallmark signs of FG including systemic manifestations of illness, crepitus, erythematous and black necrotic perineal tissue, although notably without accompanying pain. It is conceivable that the patient may have experienced or voiced complaints of pain days before hospital admission, considering the possibility of an extended period spent at home prior to being discovered. This patient's case was unique because the patient lacked the major and typical risk factors for Fournier's gangrene, such as diabetes, hypertension, and immune impairment (>50%) [20]. On the other hand, nonmodifiable risk factors, such as a patient’s age, cannot be changed. Complicating the diagnostic process was the patient's altered mental status, hindering a clear assessment of symptom onset, progression, or underlying risk factors. The patient’s medical history revealed hypertension, a significant history of cigarette smoking (40-pack years), and self-care deficits. Notably, he did not have diabetes, tested negative for human immunodeficiency virus (HIV), and lacked chronic illnesses compromising his immune system. Diabetes can increase the risk of FG because it can lead to immunosuppression, poor wound healing, and defective phagocytosis. For diabetic patients, the use of SGLT2 inhibitors can lead to the development of FG, though the exact mechanism is unknown. Prompt discontinuation of these medications is crucial to better patient outcomes. Fournier's gangrene is favored by hypertension, obesity, and chronic alcoholism.

Although the patient managed to overcome bacteremia, indicated by consistently negative blood cultures, he continued to suffer from multi-organ failure and was ultimately transferred to hospice care. Timely diagnosis and initiation of empiric antibiotic therapy are critical in mitigating patient morbidity and mortality. Research indicates a correlation between the duration from diagnosis to surgical debridement and patient survival rates [20]. Standard treatment protocols involve surgical debridement coupled with early empiric administration of broad-spectrum antibiotics, effective against both gram-positive and gram-negative bacteria, including aerobic and anaerobic species. Treatment strategies should be adjusted based on culture results and antibiotic sensitivities once available.

## Conclusions

Thorough investigation of rare, life-threatening illnesses like the one described is crucial. CT imaging aids in diagnosing FG, especially when accompanied by subcutaneous crepitation and gangrenous skin. These signs necessitate swift evaluation for infections, immunodeficiency, malignancies, and the use of certain medications. Despite lacking typical signs of immunodeficiency, the patient's history highlights risk factors such as poor physical hygiene, tobacco use, and hypertension. CT scans confirmed the diagnosis, showing extensive subcutaneous gas in various areas. Prompt antibiotic therapy and surgical intervention were administered, yet earlier detection might have improved the outcome.
